# Atlas to the Pathology of the Teeth

**Published:** 1869-12

**Authors:** 


### BIBLIOGRAPHICAL NOTICE.

*Atlas to the Pathology of the Teeth.*—Arranged and explained by the late Prof. Dr. M. Heider and Prof. Dr. C. Wedl, with drawings from nature, by Dr. C. Heitzmann, Part IV. Verlag Von Arthur Felix, Leipzig.

This number completes a very interesting and instructive work on Dental Pathology, and is illustrated by four lithograpic plates representing thirty pathological conditions, with an alphabetical register of the entire volume, Figs. 115 to 145 represent the action of pus upon dentine; absorption of hypertrophied cementum, pathological conditions of gum; deposits of lime in a thickened periosteum of the root; pathological conditions of alveolo-dental periosteum; effect of an abscess on apex of root of an incisor, also on the alveolar process of incisor and bicuspid teeth; salivary calculus; cyst in superior maxillary; transverse section of an epulis; phospor-necrosis of lower jaw; effect of an abscess in the maxillary tuberosity; malignant tumors of lower jaw, with explanations and a list of the plates in both the German and English text.

